# Dosage Error in Text, Figure, Tables, and Visual Abstract

**DOI:** 10.1001/jamanetworkopen.2024.46361

**Published:** 2024-10-18

In the Original Investigation titled “Peceleganan Spray for the Treatment of Skin Wound Infections: A Randomized Clinical Trial,”^1^ published June 11, 2024, the percent symbol (%) was used instead of the per mille symbol (‰) in the dosage for peceleganan spray throughout the article and in the Visual Abstract; the correct dosage is 2‰. This article has been corrected.
